# Potent Inducers of Endogenous Antimicrobial Peptides for Host Directed Therapy of Infections

**DOI:** 10.1038/srep36692

**Published:** 2016-11-09

**Authors:** H. Ottosson, F. Nylén, P. Sarker, E. Miraglia, P. Bergman, G. H. Gudmundsson, R. Raqib, B. Agerberth, R. Strömberg

**Affiliations:** 1 Department of Biosciences and Nutrition, Karolinska Institutet, S-14183, Huddinge, Sweden; 2 Department of Laboratory Medicine, Karolinska Institutet, S-14186, Huddinge, Sweden; 3 International Center for Diarrheal Disease Research, Bangladesh (icddr, b) Dhaka, Bangladesh; 4 Biomedical Center, University of Iceland, Reykjavik, Iceland

## Abstract

A new concept for treatment of infections is induction of our own antimicrobial peptides and the presented novel class of inducer, aroylated phenylenediamines (APDs), gives up to 20 to 30-fold induction of the human antimicrobial peptide LL-37, *in vitro*. In addition, oral administration of an APD in a rabbit model of Shigellosis resulted in recovery from the infection in a few days implying that APD’s are promising candidates for treatment of infections.

Infectious diseases, especially those caused by bacteria resistant to antibiotics, are a serious threat to society. Globally, infections caused 20% of all deaths in 2010 and among children of 1 month to 4 years of age 66–70% of all deaths were due to infections^1^. Diarrheal disease, tuberculosis and lower respiratory tract infections led to 5.4 million deaths in the same year^1^. New antibiotics have yet to match this threat, and too few novel agents have been developed in the last decades^2^,^3^. Clearly, alternative approaches to treat infections should be investigated. Host directed therapy against pathogens by boosting the expression of endogenous antimicrobial peptides (AMPs) is a novel alternative for treatment of infectious diseases. Induction of multiple AMPs would minimize the risk of microbial resistance and during the existence of mankind resistance to this “mixture” of AMPs has not been developed^4^. AMPs are crucial components of innate defences and are produced constitutively and/or at epithelial surfaces, where the initial contact with microbes takes place^5^. AMPs possess a broad activity against various pathogens, i.e. viruses, bacteria, fungi, and parasites^6^.

There are two major classes of mammalian AMPs, the defensins (α- and β-families)^7^ and the cathelicidins^8^. Besides the microbicidal activity, AMPs also modulate immune responses^9^. The human cathelicidin LL-37 can be considered a marker for antimicrobial peptide expression and this peptide has been shown to also inhibit the formation and accelerate disintegration of bacterial biofilms^10^. In addition, LL-37 was shown to work in synergy with the antibiotic azithromycin towards multi-drug resistant gram negative bacteria^11^. Recently it has been demonstrated that autophagy is activated by LL-37^12^. The expression of AMPs is downregulated by specific pathogens^13^. On the other hand, butyrate^14^ and 4-phenylbutyrate (PBA)^15^ upregulate the expression of AMPs in epithelial cells and in macrophages. In addition, 1,25-Dihydroxyvitamin D3 (vitD) enhances the expression of LL-37^16^,^17^ and in a synergistic manner with PBA^15^. Induction by butyrate or PBA resulted in pathogen elimination and improved clinical symptoms in a rabbit model of shigellosis^18^,^19^ and of enteropathogenic *E. coli* (EPEC) induced diarrhea^20^. Adjunct therapy of PBA and vitD together with conventional TB antibiotics demonstrated beneficial effects towards clinical recovery in tuberculosis^21^. These findings suggest that the concept of induction of AMPs can be a complement to antibiotics in the treatment of infections.

## Results

**Figure 1:**
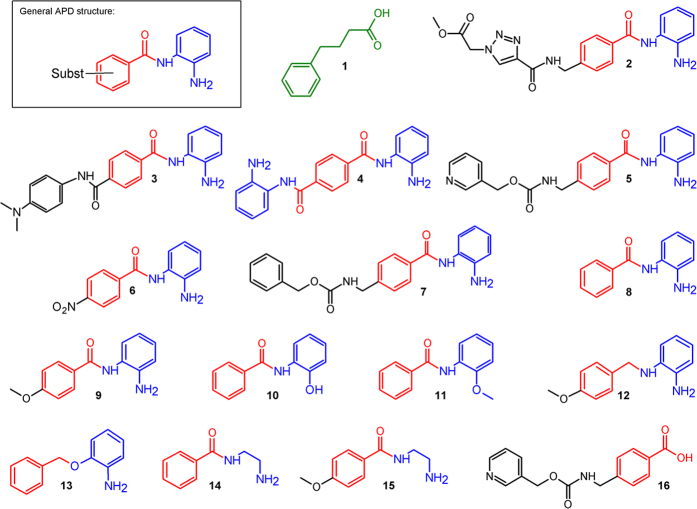
Compounds tested for their ability to induce LL-37 in the reporter cell line as shown in Figs 2, 3 and 5. Upper left corner: General structure of the potent novel inducers, aroylated phenylenediamines, APDs (phenylenediamine moieties (or less active alternatives) are presented in blue, Connecting aroyl group (or less active alternatives) in red and the substituents (Subst) of the aroyl group in black. The compound numbers are used as a reference in the text and in other figures. The previously characterized inducer PBA is number **1**.

**Figure 2:**
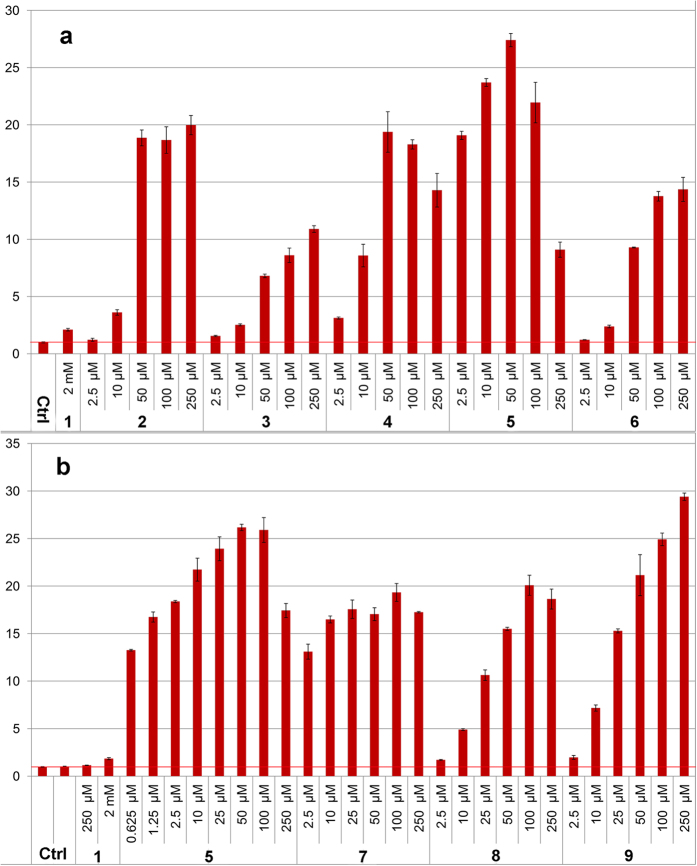
Graphs of fold induction (as luciferase activity) from assays in the MN8CampLuc reporter cell line (pre-differentiated) with the novel class of inducers represented by compounds 2–9 and also compared with induction by 2 mM PBA. The fold induction values are relative to the background from untreated cells (as indicated by the horizontal red line) and all experiments were performed in duplicate. Experiments presented in (**a**,**b**) respectively, were performed at different occasions. Bars for standard deviation are shown and all differences relative to untreated cells were statistically significant (Student’s t-test p-values were all < 0.01 except for 2.5 μM of **2** (in (**a**) p-value, 0.024)). Differences relative to 2 mM PBA (**1**) were significant for all compounds and concentrations except for the lowest concentration (2.5 μM) of **8** and **9** (in (**b**) p-values were 0.14 and 0.25 respectively). All other Student’s t-test p-values were < 0.01 except for 10 μM concentration of **6** (in (**a**) p-value 0.033).

We here present a novel class of inducers of AMPs, aroylated phenylenediamines (APDs, Fig. 1). These are considerably more potent inducers of human LL-37 than butyrate and PBA. Induction by APDs was first demonstrated in a reporter cell line (MN8CampLuc)^22^ producing a luciferase-LL-37 fusion protein. Assays in the reporter cell line with compounds **2**–**9** (Fig. 1) displayed considerably higher expression of the LL-37 fusion protein with the APD’s (Fig. 2) than with PBA (**1**) and at much lower concentration (μM range for APD’s *vs* 2 mM PBA).

Induction with pyridin-3-ylmethyl (4-((2-aminophenyl)carbamoyl)benzyl)carbamate (**5**, also called Entinostat) required the lowest concentration followed by compounds **2**, **4, 7, 8** and **9** that required a somewhat higher concentration to reach a similar fold induction (Fig. 2). The levels of induction obtained with this novel class of inducers was up to 15–20 times higher than with PBA at 100–1000 times lower concentration (Fig. 2). This was also largely independent of the differentiation stage of the cells (see Supplementary S1). The ability of the compounds to induce LL-37 expression was clearly dependent on the substitution of the aroyl group.

Some of the compounds tested are known to be histone deacetylase (HDAC) inhibitors (as are butyrate and PBA). However, earlier results show that considerably more powerful HDAC inhibitors could only induce LL-37 in the same range as PBA^22^. In addition, when induction with compound **5** was compared to that obtained with Vorinostat and Trichostatin A no direct relationship to HDAC inhibition was observed (see Supplementary S2). Although some induction is likely to occur due to HDAC inhibition the results suggest that the main mechanism for induction with APD’s is not gene activation through inhibition of histone deacetylation but through other mechanisms. We have, in a parallel study, found that compound **5** activates STAT3, which promotes transcription of the *CAMP* gene by increasing the expression of HIF-1α^23^. This seems to be one major mechanism for regulation of gene expression of LL-37, although there may also be other mechanisms involved. We have, however, generated results on how changes in APD structure affect the induction ability. The rationale for variation of the structure of the compounds arose from that **5** was the compound active at the lowest concentrations and we hypothesized that the basic structure essential for inducing ability may be the aroylated phenylenediamine core (Fig. 1), while substituents on this structure may have further effect on the activity.

**Figure 3:**
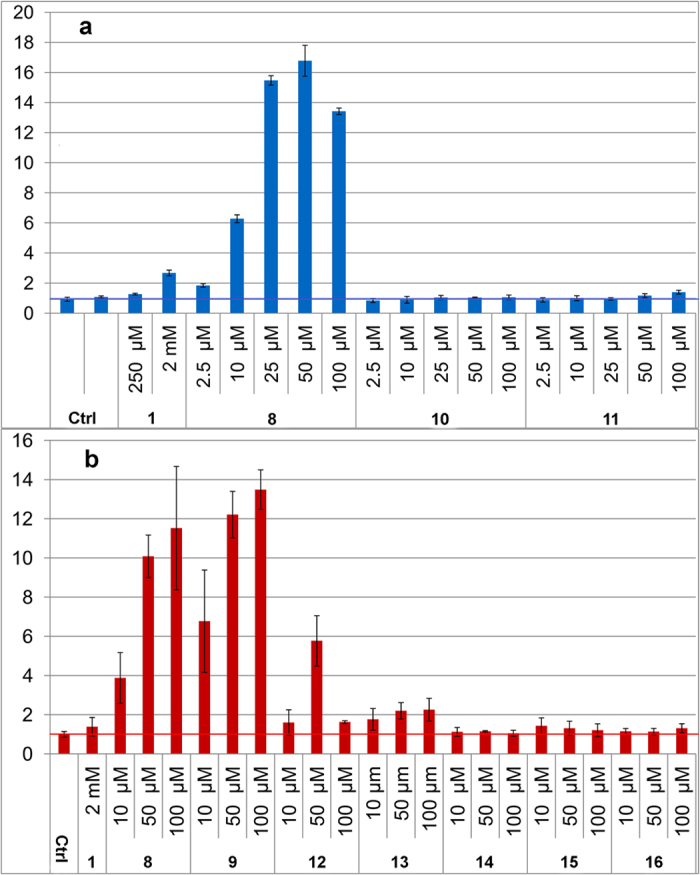
Graphs of fold induction (as luciferase activity) in the MN8CampLuc reporter cell line with compounds **12**–**16** as compared with **8** and/or **9**. (**a**) Compounds **10** and **11**, with the free amino group in the phenylenediamine moiety replaced with a hydroxyl or methoxy group, display virtually no activity as opposed to **8**. (**b**) Compound **12**, with a methylene group instead of a carbonyl attached to the one of the nitrogens of the phenylenediamine moiety, displays a significantly lower induction than with **9**. With **13**, where this nitrogen is replaced with an oxygen, an even larger difference to **8** and **9** is found. Compounds **14** and **15**, with ethylenediamine instead of phenylenediamine moieties, gave very low induction relative to **8** and **9**. The same lack of induction was also observed for **16** that completely lacks a diamine moiety. The fold induction values are relative to the control of untreated cells (as indicated by the horizontal line at a value of one) and all experiments were performed in duplicate. Experiments presented in (**a**,**b**) respectively, were performed at different occasions (**a**) not predifferentiated, (**b**) pre-differentiated). Bars for standard deviation are shown. In **a**, all differences, at the same concentrations, between compounds **8** and **10** or **11** were statistically significant (Student’s t-test p-values were all < 0.01). There was no significant difference between control and **10** or **11** (p-values 0.099–0.49) except with 10 μM of **11** (p-value 0.010). In (**b**) all differences, at the same concentrations, between compounds **12**–**15** and **8** or **9** respectively were statistically significant at 50 and 100 μM concentrations (p-values < 0.03) and at 10 μM for **14** (p-value 0.049), while at 10 μM p-values were a bit higher (0.056, 0.084, 0.052 for **12**, **13** and **15** respectively). The difference towards control was not significant at any concentration of **16** (p-values > 0.12).

**Figure 4:**
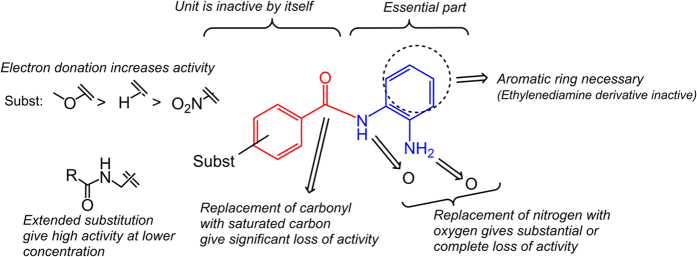
Schematic summary of relationships between structural changes in APDs and their ability to induce LL-37 in the reporter cell line.

Investigation of structure-activity relationships showed that the phenylenediamine moiety was essential for the inducing ability. Comparative studies with similar compounds revealed that one nitrogen could not be replaced by an oxygen without complete or significant loss of the inducing ability, as was evidenced by the results with compounds **10**, **11** and **13** (Fig. 3). The nitrogen also needed to be connected to an aromatic ring, thus the aromatic diamino-moiety could not be replaced by a simple aliphatic diamine as evidenced by the virtual inactivity of compounds **14** and **15** (Fig. 3b). From the weaker induction with compound **12** it is clear that the carbonyl function of the benzoic acid moiety substantially enhances induction activity (Fig. 3b). A more flexible acyl group gives substantial reduction of the inducing capability (see Supplementary S3). The substituents on **6**, **8** and **9** have increasing electron donating properties which seems to make the compound a more potent inducer (**9** > **8** > **6**, Fig. 2). Although the substitution of the benzoic acid moiety of the APDs does affect the induction potency, that moiety (compound **16**, Fig. 3b) from **5** has no inducing ability by itself, *i.e*., when not connected to phenylenediamine. A schematic summary of effects of structural changes on the inducting ability of the compounds tested is shown in Fig. 4.

**Figure 5:**
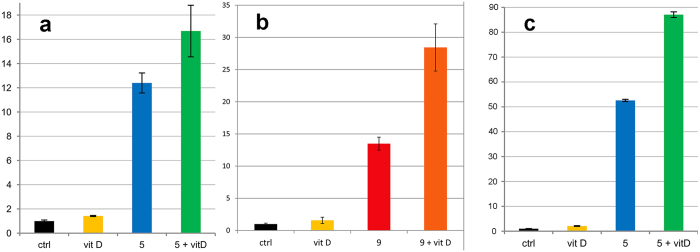
(**a,b**) Further testing of pyridin-3-ylmethyl (4-((2-aminophenyl)carbamoyl)benzyl)-carbamate (**5**, Entinostat in (**a**) 2.5 μM) and N-(2-Aminophenyl)-4-methoxybenzamide (**9** in (**b**) 100 μM), separately and in combination with 1,25-dihydroxy Vitamin D3 (VitD, 100 nM) in the MN8CampLuc reporter cell line. (**c**) Further testing of pyridin-3-ylmethyl (4-((2-aminophenyl)carbamoyl)benzyl)carbamate (**5**, 2.5 μM) separately and in combination with 1,25-dihydroxy Vitamin D3 (VitD, 100 nM) in the parental HT-29 colon epithelial cell line (RT-PCR quantification). The graphs display the benefit of the combination of the new class of inducers with VitD. Experiments presented in (**a**,**b**) respectively, were performed with different batches of cells (**a**) not predifferentiated, (**b**) pre-differentiated) performed in triplicate in (**a**) and duplicate in (**b**). Error bars represent standard deviation. In (**a**) all differences relative to control were significant (p-values < 0.01 and 0.016 for **5** + vitD) as well as the sum of vitD and **5** relative to treatment with **5** + VitD (p-value 0.021). In (**b**) all differences relative to control were significant (p-values < 0.01) as well as the sum of vitD and **9** relative to treatment with **9** + VitD (p-value 0.017). In (**c**) all differences relative to control were significant as well as the sum of vitD and **5** relative to treatment with **5** + VitD (p-values < 0.01).

Given that vitD induces LL-37 expression in synergy with PBA, we investigated if a similar effect could be found with the APDs. Stimulation of cells with a combination of an APD with vitD did indeed result in a synergistic effect in the reporter cell line, as exemplified with co-treatment with compounds **5** (Fig. 5a) and **9** (Fig. 5b). Fold induction was also substantially enhanced in the parental colon epithelial cell line, HT-29, with as low concentration as 2.5 μM of compound **5** and the synergistic effect with vitD was also evident (Fig. 5c).

**Figure 6:**
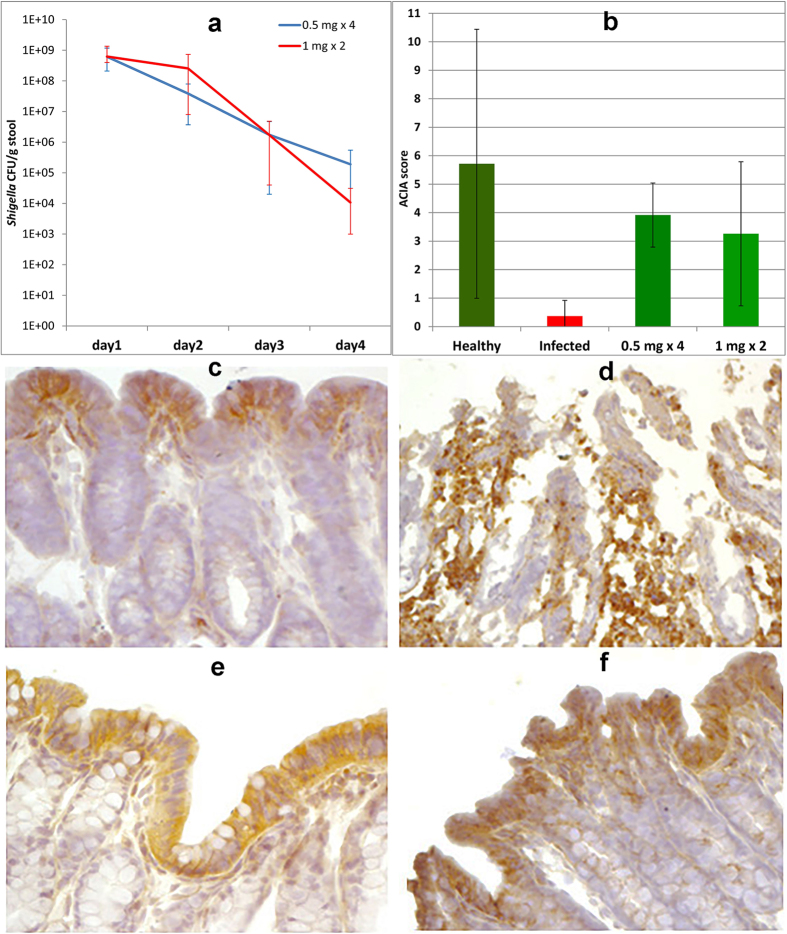
(**a**) Graph of bacterial count from stool of Shigella infected rabbits after treatment of different doses of **5** (0.5 mg twice daily for two days or 1 mg once daily for two days; 5 rabbits were used for each dose regimen but no stool was obtained from one rabbit on day 4 for the lower dose and from one rabbit given the higher dose, colonies were uncountable due to contamination, hence only results from 4 rabbits are presented for the 1 mg dose). Error bars represent spread of count values for the different rabbits. The reductions in counts from day one to four are statistically significant for both doses regimens (paired t-test p-values: 0.024 for the 0.5 mg dose and 0.018 for the 1 mg dose). (**b**) Acquired Computerized Image Analysis (ACIA)^34^ score of CAP-18 staining in rectal epithelium from healthy, infected and infected and treated rabbits (n = 5 for each group). Error bars represent standard deviation. The differences between infected and infected + treated rabbits are statistically significant (p-values < 0.01 for the 0.5 mg dose and 0.019 for the 1 mg dose). (**c**–**f**) Immunohistochemical staining of CAP-18 in sections of rabbit rectum. (**c**) Immunoreactive signals for CAP-18 (brown) in healthy rabbits were almost exclusively located in the surface epithelium. (**d**) In Shigella-infected rabbits, epithelial surface was almost devoid of CAP-18 staining and abundant CAP-18-expressing inflammatory cells were seen in the lamina propria. (**e**,**f**) Reappearance of CAP-18 staining in the surface epithelium and disappearance of CAP-18 expressing cells from the lamina propria in infected rabbits treated with 0.5 mg doses (**e**) twice daily for two days) or 1 mg doses (**f**) once daily for two days) of compound **5**.

In order to obtain proof of concept for the potential of the APDs in treatment of infections, compound **5** was used in a rabbit model of shigellosis. The outcome was that treatment of infected rabbits, by oral administration of compound **5**, resulted in a reduction of the bacterial load with about 5 log units over four days (Fig. 6a). For doses of 0.5 mg twice daily for two days and 1 mg once daily for two days, all rabbits (n = 5 in each dose group) recovered clinically over four days, whereas non-treated rabbits still had moderate (n = 1) to severe (n = 4) diarrhea (three of these rabbits died and the remaining two were sacrificed).

Three out of 5 infected rabbits that received single dose treatment with 2 mg recovered clinically and exhibited a reduced bacterial count (see Supplementary S4). However, one rabbit died and another was severely weakened without signs of improvement and was sacrificed. This may suggest that the 2 mg dose caused some toxicity, which was not observed with the lower doses. The recovery of the *Shigella* infected rabbits upon treatment with the APD was correlated to the expression of the rabbit cathelicidin CAP-18 (the rabbit ortholog to the human LL-37) in the rectal epithelium. From immunohistochemical staining of rectal tissue it was clear that infected rabbits lost expression of CAP-18 in the surface epithelium (Fig. 6b,d), which reappeared after treatment (Fig. 6b,e,f, see also Supplementary S5).

## Discussion and Conclusions

The presented results show that APDs are highly potent inducers of expression of antimicrobial peptides and we provide proof of concept that such compounds may be useful in the treatment of infections. The compound that required the lowest concentration for strong induction was compound **5**, but compound **9** gives as high induction, although at a higher concentration. On the other hand, compound **5** has a cytotoxicity similar to Trichostatin A and Vorinostat^24^, while compound **8** is considerably less toxic^25^. The nature of the substituent of the benzoic acid moiety is likely to affect toxicity as well as the induction of AMPs. We have revealed some correlations between structure and activity, but further variation of the structure is needed in order to optimize activity *vs* toxicity. Mechanistic studies^23^ and evaluation of possible side-effects should be performed in the process of selecting which compounds are most suitable for future clinical trials. It is, however, clear that there is a high potential to utilize APDs in the treatment of infections by induction of endogenous antimicrobial peptides. The direct action of the AMPs, together with additional immune-modulatory effects on innate immunity seems sufficient to clear an infection in a rabbit model. Overexpression of AMPs is likely to have an effect on gut microflora, which has been shown in a mouse model ectopically expressing human defensin 5^26^. However, it is likely that, in many cases, the main effect of inducers of AMPs, such as APDs, can be to restore an impaired mucosal barrier. APDs may be particularly advantageous in infections where a specific pathogen down-regulates expression of AMPs, which has been shown for *Shigella spp*^13^, *Neisseria gonorrhoeae*^27^ and *V. cholera*^28^. In this study, immunohistochemical staining of rabbit rectum revealed that the natural level of CAP-18 was restored rather than expressed beyond the natural level for healthy rabbits (Fig. 6b). With regard to treatment of infections by bacterial strains resistant to antibiotics we believe that APDs and the concept of induction of antimicrobial peptides may be highly beneficial. Resistance to specific AMPs has been reported^29^,^30^ but apart from acting directly on bacteria, AMPs also modulates immune responses^9^, can disintegrate biofilms^10^ and activates autophagy^12^. In addition, induction with different classes of compounds can give increased levels of a mixture of several antimicrobial components, including several AMPs.^31^ For example, in our parallel mechanistic study we have also shown induction of human β-defensin 1 by compound **5**.^23^ Additional antimicrobial components will contribute to a parallel attack on the microbe, which makes it more difficult to develop resistance. It is also likely that co-treatment of inducers of AMPs with conventional antibiotics would be highly beneficial. Synergy between AMPs and the antibiotic azithromycin towards multi-drug resistant gram negative bacteria has recently been shown^11^. An additional potential benefit of using the APDs in conjunction with conventional antibiotics is that one can expect to minimize the risk of developing bacterial resistance to classical antibiotics. Pathogens surviving antibiotic treatment could be expected to be wiped out by the induced innate immune activity. Thus, co-treatment of inducers with antibiotics would then strongly reduce the development of resistance and could provide an attractive novel approach to treat infections with multidrug resistant bacteria.

## Methods

### General methods

All reagents and solvents (analytical grade) were purchased from commercial sources and were used without further purification. The NMR spectra were collected on a Bruker DRX-400 spectrometer (400 MHz for ^1^H and 101 MHz for ^13^C) with the residual solvent signal as chemical shift reference. Mass spectra were recorded on a Micromass LCT (ESI-TOF) mass spectrometer. Compound **1**, 4-Phenylbutyric acid sodium salt (PBA, CAS#: 1716-12-7), was purchased from Tocris Bioscience (Bristol, UK), Pyridin-3-ylmethyl (4-((2-aminophenyl)carbamoyl)benzyl)-carbamate (**5**, MS-275, CAS#: 209783-80-2) and N^1^-hydroxy-N^8^-phenyloctanediamide (**17**, Vorinostat, CAS#: 149647-78-9) was purchased from LC laboratories (Woburn, MA, USA), N-(4-Methoxybenzyl)-1,2-benzenediamine (**12**, CAS#: 5729-16-8) from Fluorochem Ltd (Hadfield, UK), 2-(benzyloxy)aniline (**13**, CAS#: 20012-63-9) from Acros Organics (Geel, Belgium), and Trichostatin A (**18**, CAS#: 58880-19-6) from Sigma-Aldrich Sweden AB (Stockholm, Sweden).

### Synthesis of compounds

(Schemes are presented in the Supplementary information, S6).

### Methyl 2-[4-({4-[(2-aminophenyl)carbamoyl]benzyl}carbamoyl)-1H-1,2,3-triazol-1-yl]acetate (2) (CAS#: 1714140-86-9)

- 4-[({[1-(2-methoxy-2-oxoethyl)-1H-1,2,3-triazol-4-yl]-carbonyl}amino)methyl]benzoic acid Methyl 2-azidoacetate (100 μL, 1.03 mmol) and 4-(propiolamidomethyl)benzoic acid (221 mg, 1.09 mmol) was dissolved in methanol (10 mL). CuSO_4_*5H_2_O (135 mg, 0.54 mmol) was added followed by ascorbic acid (289 mg, 1.64 mmol) added) and a precipitate was formed. More water (5 mL) and more methanol (5 mL) was added. The reaction was stirred at room temperature overnight. EDTA-disodium salt (231 mg, 0.62 mmol) was added to the reaction mixture and then it was diluted with dichloromethane and water. The layers were separated and the water layer was extracted first with dichloromethane and second with ethyl acetate. The combined organic layers, containing almost pure product, was dried with sodium sulfate and evaporated (200 mg, 0.63 mmol, 58%). This was used without further purification in the synthesis of compound **2**. ^1^H NMR (400 MHz, DMSO-*d*_*6*_) δ ppm 12.86 (br. s., 1 H), 9.24 (t, J = 5.5 Hz, 1 H), 8.58 (s, 1 H), 7.90 (d, J = 8.1 Hz, 2 H), 7.42 (d, J = 8.1 Hz, 2 H), 5.49 (s, 2 H), 4.52 (d, J = 6.0 Hz, 2 H), 3.73 (s, 3 H); ^13^C NMR (101 MHz, DMSO-*d*_*6*_) δ ppm 167.44, 167.19, 159.71, 144.69, 142.67, 129.39, 128.04, 127.24, 52.65, 50.56, 41.82; MS (*m/z*): [M-H]^−^ calcd. for C_14_H_13_N_4_O_5_, 317. 1, found, 317.4.
- Methyl 2-[4-({4-[(2-aminophenyl)carbamoyl]benzyl}carbamoyl)-1H-1,2,3-triazol-1-yl]acetate (2) 4-[({[1-(2-methoxy-2-oxoethyl)-1H-1,2,3-triazol-4-yl]carbonyl}amino)methyl]benzoic acid (113 mg, 0.36 mmol) and N-methylmorpholine (50 μl, 0.53 mmol) was dissolved in dimethyl formamide (DMF, 10 mL) and cooled to ca −10 °C in an ice/salt bath. Isobutyl chloroformate (ca 0.05 mL, 0.4 mmol) was added. After 15 min, 1,2-phenylenediamine (68 mg, 0.63 mmol) was added. After an additional 15 min, the cooling was removed and the mixture was left at room temperature overnight. The reaction mixture was diluted with dichloromethane, extracted with HCl (1 M). The water layer was basified with NaOH (5 M) and extracted with dichloromethane. The organic layer was dried with sodium sulfate and evaporated to dryness to yield the crude product. Recrystallization from methanol yielded pure product (7 mg, 0.017 mmol, 5%). ^1^H NMR (400 MHz, DMSO-*d*_*6*_) δ ppm 9.60 (s, 1 H), 9.24 (t, J = 6.6 Hz, 1 H), 8.58 (s, 1 H), 7.93 (d, J = 8.1 Hz, 2 H), 7.44 (d, J = 8.1 Hz, 2 H), 7.16 (d, J = 7.6 Hz, 1 H), 6.97 (t, J = 7.6 Hz, 1 H), 6.78 (d, J = 8.1 Hz, 1 H), 6.59 (t, J = 7.6 Hz, 1 H), 5.49 (s, 2 H), 4.88 (br. s., 2 H), 4.53 (d, J = 6.0 Hz, 2 H), 3.73 (s, 3 H); ^13^C NMR (101 MHz, DMSO-*d*_*6*_) δ ppm 167.44, 165.13, 159.65, 143.07, 142.71, 133.15, 127.99, 127.78, 127.03, 126.62, 126.42, 123.33, 116.23, 116.10, 52.64, 50.54, 41.79; MS (*m/z*): [M + H]^+^ calcd. for C_20_H_21_N_6_O_4_, 409.2, found, 409.8.

### N^1^-(2-aminophenyl)-N^4^-((4-(dimethylamino)phenyl)amino)terephthalamide (3) (CAS#: 1714140-87-0)

Terephthaloyl chloride (1.00 g, 4.91 mmol) was dissolved in dichloromethane (30 mL) at 0 °C. N,N-dimethylbenzene-1,4-diamine (0.74 g, 5.41 mmol) and pyridine (0.40 mL, 4.95 mmol) in dichloromethane (20 mL) was added dropwise at 0 °C, the cooling was removed and the reaction mixture was left at room temperature overnight. Half of this reaction mixture was added dropwise to a solution of 1,2-phenylenediamine (1.26 g, 11.7 mmol) and pyridine (0.50 mL, 6.2 mmol) in dichloromethane (20 mL) at 0 °C. The cooling was removed and the reaction mixture was allowed to attain room temperature and was stirred for an additional 2 hrs and then diluted with dichloromethane. A precipitate that was formed in the reaction was filtered off, the filtrate was washed with water and extracted with HCl (1 M). The acidic water layer was basified with NaOH (5 M), extracted with dichloromethane. The organic layer was dried with sodium sulfate and evaporated to dryness. Recrystallization from methanol yielded pure product (245 mg, 0.654 mmol, 27%). ^1^H NMR (400 MHz, DMSO-*d*_*6*_) δ ppm 10.20 (s, 1 H), 9.90 (br. s, 1 H), 8.03–8.20 (m, 4 H), 7.62 (d, J = 8.1 Hz, 2 H), 7.20 (d, J = 7.6 Hz, 1 H), 6.98 (t, J = 7.6 Hz, 1 H), 6.80 (d, J = 7.6 Hz, 1 H), 6.74 (d, J = 8.6 Hz, 2 H), 6.61 (t, J = 7.5 Hz, 1 H), 4.98 (br. s, 2 H), 2.88 (s, 6 H); ^13^C NMR (101 MHz, DMSO-*d*_*6*_) δ ppm 164.68, 163.94, 147.39, 143.24, 137.42, 136.81, 128.66, 127.77, 127.41, 126.82, 123.11, 121.92, 116.16, 112.39, 40.42; MS (*m/z*): [M + H]^+^ calcd. for C_22_H_23_N_4_O_2_, 375.2, found, 375.4.

### N^1^,N^4^-Bis(2-aminophenyl)terephthalamide (4) (CAS#: 109702-84-3)

Terephthaloyl chloride (0.83 g, 4.09 mmol) in dichloromethane (30 mL) was added dropwise to a solution of 1,2-phenylenediamine (1.85 g, 17.11 mmol) and pyridine (1.5 mL, 18.55 mmol) in dichloromethane (60 mL) at 0 °C. The reaction mixture was allowed to attain room temperature and was stirred for an additional hour and then diluted with dichloromethane. A precipitate that was formed in the reaction was filtered off and the filtrate was washed with water, extracted with HCl (1 M). The water layer was basified with NaOH (5 M) and extracted with dichloromethane. The organic layer was dried with sodium sulfate and evaporated to dryness. Recrystallization from ethanol yielded a pure product (101 mg, 0.29 mmol, 7%). ^1^H NMR (400 MHz, DMSO-*d*_*6*_) δ ppm 9.81 (br. s., 2 H), 8.11 (br. s., 4 H), 7.20 (d, J = 7.5 Hz, 2 H), 7.00 (dd, J = 8.0, 7.2 Hz, 2 H), 6.81 (d, J = 8.0 Hz, 2 H), 6.62 (t, J = 7.3 Hz, 2 H), 4.94 (br. s., 4 H); ^13^C NMR (101 MHz, DMSO-*d*_*6*_) δ ppm 164.76, 143.28, 137.02, 127.74, 126.86, 126.72, 123.05, 116.28, 116.16; MS (*m/z*): [M + H]^+^ calcd. for C_20_H_19_N_4_O_2_, 347.2, found, 347.4.

### N-(2-Aminophenyl)-4-nitrobenzamide (6) (CAS#: 6338-73-4)

To a solution of 1,2-phenylenediamine (2.16 g, 20.0 mmol) in dichloromethane (100 mL) at room temperature was added p-nitrobenzoyl chloride (1.86 g, 10.2 mmol) and the mixture was stirred overnight. A thick precipitate was formed and was filtered off (contains both mono and dinitrobenzoyl product). The solution was extracted with HCl (1 M). More precipitate was formed and filtered off (mostly mono nitrobenzoyl-product). The water layer was basified with NaOH (ca 5 M) and extracted with dichloromethane, washed with water, dried, evaporated. The fractions containing mainly N-(2-Aminophenyl) 4-nitrobenzamide were combined and recrystallization from methanol gave a pure product (67.5 mg pure, 0.262 mmol, 3%). ^1^H NMR (400 MHz, DMSO-*d*_*6*_) δ ppm 9.94 (s, 1 H), 8.35 (d, J = 8.6 Hz, 2 H), 8.22 (d, J = 8.6 Hz, 2 H), 7.18 (d, J = 7.6 Hz, 1 H), 7.00 (t, J = 7.6 Hz, 1 H), 6.79 (d, J = 7.6 Hz, 1 H), 6.60 (t, J = 7.6 Hz, 1 H), 4.98 (s, 2 H); ^13^C NMR (101 MHz, DMSO-*d*_*6*_) δ ppm 163.80, 149.04, 143.37, 140.44, 129.30, 126.90, 123.38, 122.48, 116.07, 115.97; MS (*m/z*): [M + H]^+^ calcd. for C_13_H_12_N_3_O_3_, 258.1, found, 258.5.

### Benzyl {4-[(2-aminophenyl)carbamoyl]benzyl}carbamate (7) (CAS#: 209783-81-3)

- 4-({[(benzyloxy)carbonyl]amino}methyl)benzoic acid (CAS#: 58933-52-1) 4-(Aminomethyl)benzoic acid (4.53 g, 30.0 mmol) was dissolved in acetone (50 mL), sodium hydrogen carbonate (aqueous saturated (*aq*, sat.), 50 mL), and water (50 mL). After cooling to 0 °C and adding of ice (ca 10 mL) to the reaction mixture, benzyl chloroformate (4.5 mL, 31.5 mmol) in acetone (25 mL) was added dropwise. The mixture was allowed to attain room temperature and then stirred overnight. The mixture was diluted with water and washed with dichloromethane. The water layer was acidified with HCl (1 M) and extracted with ethyl acetate. The organic layer was basified with NaOH (*aq*) and a precipitate was formed. This was filtered off, washed with acetone and dichloromethane. Recrystallization from methanol yielded pure product as the sodium salt (2.76 g, 9.0 mmol, 30%). ^1^H NMR (400 MHz, DMSO-*d*_6_) δ ppm 7.90 (d, J = 8.1 Hz, 3 H), 7.26–7.43 (m, 8 H), 5.05 (s, 2 H), 4.28 (d, J = 6.0 Hz, 2 H); ^13^C NMR (101 MHz, DMSO-*d*_*6*_) δ ppm 167.39, 156.72, 145.24, 137.32, 129.69, 129.45, 128.69, 128.16, 128.01, 127.31, 65.79, 43.77; MS (*m/z*): [M-H]^−^ calcd. for C_16_H_14_NO_4_, 284.1, found, 284.4.
- Benzyl {4-[(2-aminophenyl)carbamoyl]benzyl}-carbamate (7) 4-({[(benzyloxy)carbonyl]amino}methyl)benzoic acid (313 mg, 1.02 mmol) was evaporated with toluene and then dissolved in DMF (15 mL). N-Methylmorpholine (132 μl 1.20 mmol) was added followed by isobutyl chloroformate (0.17 mL, 1.3 mmol). The mixture was stirred at room temperature for about 15 min whereafter 1,2-phenylendiamine (173 mg, 1.60 mmol) was added. The mixture was stirred at room temperature overnight and then evaporated to yield the crude product as oil. The oil was dissolved in dichloromethane and crystals was formed, filtered off and washed with dichloromethane. Recrystallization from methanol gave pure product (66 mg, 0.176 mmol, 17%). ^1^H NMR (400 MHz, DMSO-*d*_*6*_) δ ppm 9.63 (br. s, 1 H), 7.91 (t, J = 5.5 Hz, 1 H), 7.86–7.98 (m, 3 H), 7.28–7.46 (m, 7 H), 7.18 (d, J = 7.6 Hz, 1 H), 6.97 (t, J = 7.6 Hz, 1 H), 6.79 (d, J = 7.6 Hz, 1 H), 6.61 (t, J = 7.6 Hz, 1 H), 5.06 (s, 2 H), 4.91 (br. s., 2 H), 4.29 (d, J = 6.0 Hz, 2 H); ^13^C NMR (101 MHz, DMSO-*d*_*6*_) δ ppm 165.13, 156.43, 143.26, 143.07, 137.13, 133.19, 128.38, 127.83, 127.76, 126.74, 126.66, 126.45, 123.37, 116.29, 116.16, 65.46, 43.60; MS (*m/z*): [M + H]^+^ calcd. for C_22_H_22_N_3_O_3_, 376.2, found, 376.4.

### N-(2-Aminophenyl)benzamide (8) (CAS#: 4424-17-3)

To a solution of 1,2-phenylenediamine (1.8 g, 16.6 mmol) in dichloromethane (40 mL) at room temperature was added potassium carbonate (1.28 g, 9.26 mmol) and benzoyl chloride (1.00 mL, 8.62 mmol). After about 30 s a precipitate was formed. The mixture was stirred for 1 hr, the precipitate was filtered off and washed with dichloromethane. The filtrate was washed with water and then extracted with HCl (1 M). Some precipitate was formed, while extracting, and was filtered off. The water layer was basified with NaOH (*aq*) and extracted with dichloromethane. The dichloromethane layer was dried with sodium sulfate and evaporated. The resulting solid was recrystallized from diethyl ether. Several recrystallizations yielded a pure sample (423 mg, 1.99 mmol, 23%). ^1^H NMR (400 MHz, chloroform-*d*) δ ppm 7.92 (d, J = 7.6 Hz, 2 H), 7.88 (br. s, 1 H), 7.57 (t, J = 7.6 Hz, 1 H), 7.50 (t, J = 7.6 Hz, 2 H), 7.34 (d, J = 8.1 Hz, 1 H), 7.11 (td, J = 7.8, 1.1 Hz, 1 H), 6.82–6.89 (m, 2 H), 3.88 (br. s., 2 H); ^13^C NMR (101 MHz, DMSO-*d*_*6*_) δ ppm 166.08, 140.92, 134,47, 132.20, 129.04, 127.50, 127,53, 125.42, 124.86, 120.08, 118.68; MS (*m/z*): [M + H]^+^ calcd. for C_13_H_13_N_2_O, 213.1, found, 213.5.

### N-(2-Aminophenyl)-4-methoxybenzamide (9) (CAS#: 103517-57-3)

To a solution of 1,2-phenylenediamine (1.30 g, 12.0 mmol) in dichloromethane (30 mL) at room temperature was added potassium carbonate (1.4 g, 10.0 mmol) and 4-methoxybenzoyl chloride (1.57 g, 9.2 mmol) in dichloromethane (10 mL). The reaction mixture was stirred at room temperature for 1 hr and then diluted with dichloromethane and water to give two clear phases, which were separated. The dichloromethane layer was extracted with HCl (1 M) and a precipitate was formed. This precipitate was filtered off, washed with dichloromethane and some ethanol to give crude product as the hydrochloride salt. The precipitate was recrystallized from ethanol to yield the product as the hydrochloride (307 mg, 1.1 mmol, 12%). The filtrate was washed with water, and then it was extracted with HCl (1 M). This latter water layer was basified with NaOH and extracted with dichloromethane. The dichloromethane layer was dried with sodium sulfate and evaporated to yield crude N-(2-Aminophenyl) 4-methoxybenzamide as the free amine. The resulting solid was recrystallized from ethanol yielded pure product (176 mg, 0.7 mmol, 8%). ^1^H NMR (400 MHz, DMSO-*d*_*6*_) δ ppm 10.45 (s, 1 H), 8.13 (d, J = 9.1 Hz, 2 H), 7.59 (d, J = 8.1 Hz, 1 H), 7.50 (d, J = 8.0 Hz, 1 H), 7.40 (t, J = 8.0 Hz, 1 H), 7.32 (t, J = 8.0 Hz, 1 H), 7.07 (d, J = 8.6 Hz, 2 H), 3.85 (s, 3 H); ^13^C NMR (101 MHz, DMSO-*d*_*6*_) δ ppm 165.21, 162.26, 131.59, 130.10,, 127.65, 127.22, 127.17, 126.38, 125.68, 123.76, 113.63, 55.50; MS (*m/z*): [M + H]^+^ calcd. for C_14_H_15_N_2_O_2_, 243.1, found, 243.5.

### N-×(2-Hydroxyphenyl) benzamide (10) (CAS#: 3743-70-2)

To a solution of 2-aminophenol (0.59 g, 5.4 mmol) in dichloromethane (10 mL) and pyridine (0.50 mL, 0.62 mmol), at 0 °C was added benzoyl chloride (0.65 mL, 5.6 mmol) in dichloromethane (10 mL), and the mixture was stirred for 3 hrs. The reaction mixture was diluted with dichloromethane and washed with sodium hydrogen carbonate (*aq*, sat.), dried with sodium sulfate and evaporated to dryness to give a crude mixture of N-(2-hydroxyphenyl)benzamide and 2-(benzoylamino)phenyl benzoate. Recrystallized from methanol also gave a mixture of these two compounds.

A mixture of crude mono- and di-benzoyl derivatives (400 mg, ca 7 mmol) was dissolved in methanol (10 mL) and 1 mL 30% sodium methoxide in methanol was added. When no more 2-(benzoylamino)phenyl benzoate could be detected by thin layer chromatography (TLC), the reaction was neutralized with acetic acid and evaporated to dryness. The crude product was dissolved in dichloromethane and washed with sodium hydrogen carbonate (*aq*, sat.) dried with sodium sulfate and evaporated to dryness to give crude N-(2-hydroxyphenyl)benzamide. Several recrystallizations from methanol gave a pure product (69 mg, 0.31 mmol). ^1^H NMR (400 MHz, DMSO-*d*_*6*_) δ ppm 9.74 (s, 1 H), 9.52 (s, 1 H), 7.98 (d, J = 7.6 Hz, 2 H), 7.70 (dd, J = 8.00, 1.4 Hz, 1 H), 7.60 (t, J = 7.3 Hz, 1 H), 7.53 (t, J = 7.6 Hz, 2 H), 7.04 (td, J = 7.7, 1.4 Hz, 1 H), 6.93 (d, J = 8.1 Hz, 1 H), 6.84 (t, J = 7.6 Hz, 1 H); ^13^C NMR (101 MHz, DMSO-*d*_*6*_) δ ppm 165.25, 149.32, 134.38, 131.65, 128.50, 127.48, 125.87, 125.68, 124.09, 119.03, 115.99; MS (*m/z*): [M-H]^−^ calcd. for C_13_H_10_NO_2_, 212.1, found, 212.5.

### N-(2-methoxyphenyl)benzamide (11) (CAS#: 5395-00-6)

To a stirred mixture of anisidine (1.128 mL, 10.0 mmol) and potassium carbonate (1.45 g, 10.5 mmol) in dichloromethane (40 mL), benzoylchloride (1.16 mL, 10.0 mmol) was added. After ca 30 s a precipitate was formed. The mixture was left at room temperature overnight and then diluted with dichloromethane, washed with water, HCl (1 M) and sodium hydrogen carbonate (*aq*, sat.). The organic layer was dried with sodium sulfate and evaporated. Silica gel chromatography (hexane: ethyl acetate: dichloromethane gradient) yielded pure N-(2-methoxyphenyl)benzamide (2.2 g, 9.7 mmol, 97%) as an oil. ^1^H NMR (400 MHz, chloroform-*d*) δ ppm 8.57 (br. s, 1 H), 8.55 (dd, J = 8.1, 1.5 Hz, 1 H), 7.91 (dd, J = 7.6, 1.5 Hz, 2 H), 7.47–7.60 (m, 3 H), 7.10 (td, J = 7.6, 1.5 Hz, 1 H), 7.04 (td, J = 7.7, 1.4 Hz, 1 H), 6.94 (d, J = 7.6 Hz, 1 H), 3.94 (s, 3 H); ^13^C NMR (101 MHz, chloroform-*d*) δ ppm 165.49, 148.39, 135.59, 131.92, 128.99, 128.05, 127.29, 124.11, 121.46, 120.09, 110.18, 56.06; MS (*m/z*): [M + H]^+^ calcd. for C_14_H_14_NO_2_, 228.1, found, 228.5.

### N-(2-aminoethyl)-benzamide (14) (CAS#: 1009-17-2)

Ethylenediamine (2.00 mL, 30.0 mmol) was dissolved in dichloromethane (100 mL) benzoyl chloride (1.16 mL, 10.0 mmol) in dichloromethane (20 mL) added dropwise at 0 °C. A white precipitate was formed and the reaction mixture was left at room temperature overnight. The precipitate was filtered off and washed with dichloromethane. The filtrate was evaporated to give a mixture of N-(2-aminoethyl)-benzamide and N,N’-ethane-1,2-diyldibenzamide as an oil. The oil was triturated with ethyl acetate and a precipitate was formed, which was filtered off. The filtrate was concentrated and subsequently purified on silica gel chromatography (hexane:dichloromethane:methanol gradient) to give a not fully pure product (544 mg, 3.3 mmol, 33%). Further purification with repeated triturations/crystallizations from dichloromethane and methanol gave a pure sample of N-(2-aminoethyl)-benzamide (69 mg, 0.42 mmol, 4%). ^1^H NMR (400 MHz, DMSO-*d*_*6*_) δ ppm 8.41 (br, 1 H), 7.83–7.87 (m, 2 H), 7.41–7.54 (m, 3 H), 3.27 (q, J = 6.6 Hz, 2 H), 2.69 (t, J = 6.5 Hz, 2 H), 1.86 (br. s, 2 H); ^13^C NMR (101 MHz, DMSO-*d*_*6*_) δ ppm 166.34, 134.69, 130.96, 128.17, 127.15, 43.02, 41.30; MS (*m/z*): [M-H]^−^ calcd. for C_9_H_11_N_2_O, 163.1, found, 163.5.

### N-(2-aminoe°thyl)-4-methoxybenzamide (15) (CAS#: 65136-87-0)

Ethylenediamine (2.0 mL, 30.0 mmol) was added to a solution of trifluoroacetic acid (2.31 mL, 30.2 mmol) in dichloromethane (20 mL). Methanol was added until a clear solution was fomed (20 mL). This solution was cooled in an ice bath, whereupon 4-methoxybenzoyl chloride (900 mg, 1.47 mmol) in dichloromethane (10 mL) was added dropwise. The reaction mixture was left at room temperature overnight. The reaction mixture was diluted with dichloromethane and extracted with hydrochloric acid (1 M). The water layer was basified with sodium hydroxide (*aq*) and extracted with dichloromethane to yield a crude mixture of N-(2-aminoethyl)-4-methoxybenzamide and N,N’-ethane-1,2-diylbis(4-methoxybenzamide). Purification was performed by repeated triturations/crystallizations from dichloromethane and methanol. A final crystallization from methanol and a small amount of trifluoroacetic acid yielded pure N-(2-aminoethyl)-4-methoxybenzamide as the trifluoroacetic acid salt (346 mg, 1.12 mmol, 21%). ^1^H NMR (400 MHz, DMSO-*d*_6_) δ ppm 8.53 (t, J = 5.54 Hz, 1 H), 7.94 (br. s., 3 H), 7.85 (d, J = 9.1 Hz, 2 H), 7.00 (d, J = 9.1 Hz, 2 H), 3.81 (s, 3 H), 3.49 (td, J = 6.3, 5.5 Hz, 2 H), 2.99 (t, J = 6.3 Hz, 2 H); ^13^C NMR (101 MHz, DMSO-*d*_*6*_) δ ppm 166.41, 161.70, 158.48 (q, *J* = 31.00 Hz), 129.17, 126.28, 117.21 (q, *J* = 299.40 Hz), 113.45, 55.35, 38.73, 37.17; MS (*m/z*): [M + H]^+^ calcd. for C_10_H_15_N_2_O_2_, 195.1133, found, 195.5633.

### 4-((((pyridin-3-ylmethoxy)carbonyl)amino)methyl)benzoic acid (16) (CAS#: 241809-79-0)

#### Prepared according to the procedure described by Gediya L., *et al*

^1^H NMR (400 MHz, DMSO-*d*_6_) δ ppm 12.89 (br. s, 1 H), 8.59 (br. s., 1 H), 8.53 (d, J = 3.8 Hz, 1 H), 7.95 (t, J = 5.9 Hz, 1 H), 7.90 (d, J = 8.1 Hz, 2 H), 7.78 (d, J = 7.8 Hz, 1 H), 7.41 (dd, J = 7.5, 5.0 Hz, 1 H), 7.36 (d, J = 8.1 Hz, 2 H), 5.10 (s, 2 H), 4.28 (d, J = 6.0 Hz, 2 H); ^13^C NMR (101 MHz, DMSO-*d*_*6*_) δ ppm 167.15, 156.25, 149.13, 149.10, 144.76, 135.76, 132.65, 129.39, 129.36, 126.99, 123.52, 63.27, 43.63; MS (*m/z*): [M-H]^−^ calcd. for C_15_H_13_N_2_O_4_, 285.1, found, 285.4^32^.

### N^1^-(2-aminophenyl)-N^5^-(4-(dimethylamino)phenyl)glutaramide (19) (CAS#: 1714140-88-1)

- 5-{[4-(dimethylamino)phenyl]amino}-5-oxopentanoic acid To a stirred solution of N,N-dimethylbenzene-1,4-diamine (1.67 g, 12.3 mmol) in dichloromethane (30 mL) pyridine (0.8 mL, 10.0 mmol) was added, followed by glutaric anhydride (1.14g, 10.0 mmol) in dichloromethane(10 mL). The reaction was stirred at room temperature overnight. The mixture was diluted with dichloromethane and extracted with sodium hydrogen carbonate (aq, sat). The water layer was adjusted to ca pH 5 with HCl and was extracted with dichloromethane and ethyl acetate. The combined organic layers were dried with sodium sulfate and evaporated. Recrystallization from methanol yielded a pure product (454 mg, 1.81 mmol, 18%). ^1^H NMR (400 MHz, DMSO-*d*_6_) δ ppm 12.05 (br, 1 H), 9.56 (s, 1 H), 7.38 (d, J = 9.1 Hz, 2 H), 6.66 (d, J = 9.1 Hz, 2 H), 2.82 (s, 6 H), 2.27 (q, J = 7.5 Hz, 4 H), 1.79 (quin, J = 7.3 Hz, 2 H); ^13^C NMR (101 MHz, DMSO-*d*_*6*_) δ ppm 174.19, 169.84, 146.93, 129.22, 120.58, 112.68, 40.54, 35.24, 33.06, 20.63; MS (*m/z*): [M-H]^−^ calcd. for C_13_H_17_N_2_O_3_, 249.1, found, 249.5.
- N1-(2-aminophenyl)-N5-(4-(dimethylamino)phenyl)-glutaramide (**19**) N-Methylmorpholine (138 μL, 1.26 mmol)) was added to a stirred solution of 5-{[4-(dimethylamino)phenyl]amino}-5-oxopentanoic acid (313 mg, 1.25 mmol) in dichloromethane (20 mL) at room temperature. After 30 min, isobutyl chloroformate (0.2 mL, 1.5 mmol) was added and after 10 min followed by 1,2-phenylenediamine (143 mg, 1.32 mmol) in dichloromethane (5 mL). The reaction was left at room temperature overnight, diluted with dichloromethane and water. The mixture was extracted with HCl (1 M). The water layer was basified with NaOH (5 M) and extracted with dichloromethane. The organic layer was evaporated to give the crude product. Recrystallization from ethanol yielded a pure product (55.1 mg, 0.162 mmol, 13%). ^1^H NMR (400 MHz, DMSO-*d*_*6*_) δ ppm 9.60 (s, 1 H), 9.10 (s, 1 H), 7.41 (d, J = 9.1 Hz, 2 H), 7.18 (d, J = 7.6 Hz, 1 H), 6.89 (t, J = 7.6 Hz, 1 H), 6.71 (d, J = 8.1 Hz, 1 H), 6.67 (d, J = 9.1 Hz, 2 H), 6.53 (t, J = 7.3 Hz, 1 H), 4.88 (br. s, 2 H), 2.83 (s, 6 H), 2.37 (t, J = 7.3 Hz, 2 H), 2.32 (t, J = 7.6 Hz, 2 H), 1.89 (t, J = 7.3 Hz, 2 H); ^13^C NMR (101 MHz, DMSO-*d*_*6*_) δ ppm 170.75, 169.96, 146.86, 141.85, 129.29, 125.66, 125.33, 123.49, 120.53, 116.09, 115.80, 112.65, 40.53, 35.50, 35.05, 21.35; MS (*m/z*): [M + H]^+^ calcd. for C_19_H_25_N_4_O_2_, 341.2, found, 341.5.

### Cell assays

MN8CampLuc cells were handled according to Nylén, *et al*.^22^, with the following exception when pre-differention of cells were performed before induction: Cell seeding was performed in medium where glucose was exchanged for galactose (5 mg/ml), which is known to promote differentiation in colon epithelial cells^33^. Cells were then allowed to grow for 72 hours before stimulation with test compounds. RT-PCR experiments upon induction of the HT-29 parental cell line were performed according to Nylén, *et al*.^22^. Results were normalized by the expression of the 18 s rRNA housekeeping gene. There is some variability from experiment to experiment with this assay, especially with different batches of cells (even as much as up to 50%) but the relative large differences and trends remain the same for different experiments. In general, we also include at least two positive controls to compare with for each experiment. Statistical analysis was performed by Student’s t-test, one-sided, on duplicates with an alpha of 0.05, unless otherwise stated. Only p-values higher than 0.01 are specified.

### Animal experiments

The study was approved by the Animal Experimentation Ethics Committee (AEEC) of the International Centre for Diarrhoeal Disease Research, Bangladesh (icddr,b) (Research protocol # 2013–075). The methods were carried out in accordance with relevant guidelines. Based on the recommendations in the Guide for the Care and Use of Laboratory Animals of the National Institutes of Health (NIH), icddr,b has developed its own rules and guidelines. Inbred New Zealand white rabbits of either sex weighing 1.7–2.0 kg were maintained in the animal resource facilities of icddr,b. Healthy rabbits free of enteric pathogens e.g. *Salmonella, Shigella, Vibrio cholera*e and Coccidia were studied. A nonsurgical rabbit model of shigellosis was used as described^18^. Suspension of *S. flexneri* 2a [10^9^ cfu] was given via a sterile feeding tube to each rabbit to develop shigellosis. After development of dysenteric symptoms (usually within 24 h of inoculation), rabbits were treated with 0.5 mg entinostat twice a day for 2 days (n = 5) or 1 mg entinostat once a day for 2 days (n = 5) or left untreated (n = 5). Bacterial suspension and entinostat was given in 7-ml of normal saline. All rabbits were observed for clinical outcome (temperature, body weight, activity level and food and water intake) throughout the study period. Bacterial load in stool was quantified by plating serial dilutions of stool onto MacConkey Agar (from the icddrb media facility) plates and colonies were counted after an overnight incubation at 37 °C. The results were expressed as colony forming units (CFU) per gram of stool. Rabbits were sacrificed with an overdose of intravenous sodium pentobarbital (66 mg/kg body weight; Sigma). Infected untreated rabbits were sacrificed when the sufferings seemed intolerable, usually on the 2^nd^ day. Tissue biopsies of rectum were collected and fixed in 10% buffered formalin, cryoprotected by infiltrating in sucrose solution followed by snap freezing in liquid nitrogen. Frozen tissues were cut into 5 micron thick sections and Immunohistochemical staining for CAP-18 was performed by using the affinity purified chicken polyclonal antibody (Innovagen, Lund, Sweden) as described^18^. Immunohistochemical staining of CAP-18 *in situ* was analyzed by using the image analysis system Quantimate Q550 (Leica Microsystems GmbH, Wetzlar, Germany). Quantification of CAP-18 staining in epithelium of each tissue section was performed at 400x magnification and the results were given as Acquired Computerized Image Analysis (ACIA) score^34^. Expression of the CAP-18 protein/peptide in infected and infected-treated rabbits was compared with healthy untreated rabbits (n = 5). Statistical analysis was performed by Student’s t-test (n = 5, *p < 0.05, one-sided, paired for the bacterial count from Shigella infection, and unpaired for the ACIA-score). Only p-values higher than 0.01 are specified.

## Additional Information

**How to cite this article**: Ottosson, H. *et al*. Potent Inducers of Endogenous Antimicrobial Peptides for Host Directed Therapy of Infections. *Sci. Rep*. **6**, 36692; doi: 10.1038/srep36692 (2016).

**Publisher’s note:** Springer Nature remains neutral with regard to jurisdictional claims in published maps and institutional affiliations.

## Supplementary Material

Supplementary Information

## References

[b1] Lozano R. . Global and regional mortality from 235 causes of death for 20 age groups in 1990 and 2010: a systematic analysis for the Global Burden of Disease Study 2010. Lancet 380, 2095–2128 (2012). 23245604 10.1016/S0140-6736(12)61728-0 PMC10790329

[b2] Spellberg B. . The Epidemic of Antibiotic-Resistant Infections: A Call to Action for the Medical Community from the Infectious Diseases Society of America. Clin. Infect. Dis. 46, 155–164 (2008). 18171244 10.1086/524891

[b3] Boucher H. W. . Bad Bugs, No Drugs: No ESKAPE! An Update from the Infectious Diseases Society of America. Clin. Infect. Dis. 48, 1–12 (2009). 19035777 10.1086/595011

[b4] Marr A. K., Gooderham W. J. & Hancock R. E. Antibacterial peptides for therapeutic use: obstacles and realistic outlook. Curr. Opin. Pharmacol. 6, 468–472 (2006). 16890021 10.1016/j.coph.2006.04.006

[b5] Cederlund A., Gudmundsson G. H. & Agerberth B. Antimicrobial peptides important in innate immunity. FEBS J. 278, 3942–3951 (2011). 21848912 10.1111/j.1742-4658.2011.08302.x

[b6] Zasloff M. Antimicrobial peptides of multicellular organisms. Nature 415, 389–395 (2002). 11807545 10.1038/415389a

[b7] Ganz T. Defensins: antimicrobial peptides of innate immunity. Nat. Rev. Immunol. 3, 710–720 (2003). 12949495 10.1038/nri1180

[b8] Zaiou M. & Gallo R. L. Cathelicidins, essential gene-encoded mammalian antibiotics. J. Mol. Med. (Berl). 80, 549–561 (2002). 12226737 10.1007/s00109-002-0350-6

[b9] Hancock R. E. W. & Sahl H.-G. Antimicrobial and host-defense peptides as new anti-infective therapeutic strategies. Nat. Biotechnol. 24, 1551–1557 (2006). 17160061 10.1038/nbt1267

[b10] Overhage J. . Human host defense peptide LL-37 prevents bacterial biofilm formation. Infect. Immun. 76, 4176–4182 (2008). 18591225 10.1128/IAI.00318-08 PMC2519444

[b11] Lin L. . Azithromycin Synergizes with Cationic Antimicrobial Peptides to Exert Bactericidal and Therapeutic Activity Against Highly Multidrug-Resistant Gram-Negative Bacterial Pathogens. EBioMedicine 2, 690–698 (2015). 26288841 10.1016/j.ebiom.2015.05.021 PMC4534682

[b12] Rekha R. S. . Phenylbutyrate induces LL-37-dependent autophagy and intracellular killing of Mycobacterium tuberculosis in human macrophages. Autophagy 11, 1688–1699 (2015). 26218841 10.1080/15548627.2015.1075110 PMC4590658

[b13] Islam D. . Downregulation of bactericidal peptides in enteric infections: a novel immune escape mechanism with bacterial DNA as a potential regulator. Nat. Med. 7, 180–185 (2001). 11175848 10.1038/84627

[b14] Schauber J. . Expression of the cathelicidin LL-37 is modulated by short chain fatty acids in colonocytes: relevance of signalling pathways. Gut 52, 735–741 (2003). 12692061 10.1136/gut.52.5.735 PMC1773650

[b15] Steinmann J., Halldórsson S., Agerberth B. & Gudmundsson G. H. Phenylbutyrate induces antimicrobial peptide expression. Antimicrob. Agents Chemother. 53, 5127–5133 (2009). 19770273 10.1128/AAC.00818-09 PMC2786349

[b16] Wang T.-T. . Cutting edge: 1,25-dihydroxyvitamin D3 is a direct inducer of antimicrobial peptide gene expression. J. Immunol. 173, 2909–2912 (2004). 15322146 10.4049/jimmunol.173.5.2909

[b17] Gombart A. F., Borregaard N. & Koeffler H. P. Human cathelicidin antimicrobial peptide (CAMP) gene is a direct target of the vitamin D receptor and is strongly up-regulated in myeloid cells by 1,25-dihydroxyvitamin D3. FASEB J. 19, 1067–1077 (2005). 15985530 10.1096/fj.04-3284com

[b18] Raqib R. . Improved outcome in shigellosis associated with butyrate induction of an endogenous peptide antibiotic. Proc. Natl. Acad. Sci. USA 103, 9178–9183 (2006). 16740661 10.1073/pnas.0602888103 PMC1482586

[b19] Sarker P. . Phenylbutyrate counteracts Shigella mediated downregulation of cathelicidin in rabbit lung and intestinal epithelia: a potential therapeutic strategy. PLoS One 6, e20637 (2011). 21673991 10.1371/journal.pone.0020637 PMC3108617

[b20] Al-Mamun A. . Treatment with phenylbutyrate in a pre-clinical trial reduces diarrhea due to enteropathogenic Escherichia coli: link to cathelicidin induction. Microbes Infect. 15, 939–950 (2013). 24016414 10.1016/j.micinf.2013.08.007

[b21] Mily A. . Significant Effects of Oral Phenylbutyrate and Vitamin D Adjunctive Therapy in Pulmonary Tuberculosis: A Randomized Controlled Trial. PLoS One 10, e0138340 (2015). 26394045 10.1371/journal.pone.0138340 PMC4578887

[b22] Nylén F. . Boosting innate immunity: development and validation of a cell-based screening assay to identify LL-37 inducers. Innate Immun. 20, 364–376 (2014). 23884095 10.1177/1753425913493338

[b23] Miraglia E., . Entinostat up-regulates the CAMP gene encoding LL-37 via activation of STAT3 and HIF-1α transcription factors. Sci Rep. 6, 33274 (2016). 27633343 10.1038/srep33274 PMC5025742

[b24] Cody J. J., Markert J. M. & Hurst D. R. Histone deacetylase inhibitors improve the replication of oncolytic herpes simplex virus in breast cancer cells. PLoS One 9, e92919 (2014). 24651853 10.1371/journal.pone.0092919 PMC3961437

[b25] Bressi J. C. . Exploration of the HDAC2 foot pocket: Synthesis and SAR of substituted N-(2-aminophenyl)benzamides. Bioorg. Med. Chem. Lett. 20, 3142–3145 (2010). 20392638 10.1016/j.bmcl.2010.03.091

[b26] Salzman N. H. . Enteric defensins are essential regulators of intestinal microbial ecology. Nat Immunol. 11, 76–83 (2010). 19855381 10.1038/ni.1825 PMC2795796

[b27] Bergman P. . Neisseria gonorrhoeae downregulates expression of the human antimicrobial peptide LL-37. Cell Microbiol. 7, 1009–1017 (2005). 15953032 10.1111/j.1462-5822.2005.00530.x

[b28] Chakraborty K. . Bacterial exotoxins downregulate cathelicidin (hCAP-18/LL-37) and human beta-defensin 1 (HBD-1) expression in the intestinal epithelial cells. Cell Microbiol. 10, 2520–2537 (2008). 18717821 10.1111/j.1462-5822.2008.01227.x

[b29] Lofton H., Pränting M., Thulin E. & Andersson D. I. Mechanisms and Fitness Costs of Resistance to Antimicrobial Peptides LL-37, CNY100HL and Wheat Germ Histones. Plos One, 8, e68875 (2013). 23894360 10.1371/journal.pone.0068875 PMC3720879

[b30] Ryan L. . Anti-antimicrobial Peptides. Folding-mediated host defense antagonists. J. Biol. Chem. 288, 20162–20172 (2013). 23737519 10.1074/jbc.M113.459560 PMC3711284

[b31] Wang G. Human Antimicrobial Peptides and Proteins. Pharmaceuticals, 7, 545–594 (2014). 24828484 10.3390/ph7050545 PMC4035769

[b32] Gediya L., Belosay A., Khandelwal A., Purushottamachar P. & Njar V. C. O. Improved Synthesis of Histone Deacetylase Inhibitors (HDIs) (MS-275 and CI-994) and Inhibitory Effects of HDIs Alone or in Combination with RAMBAs or Retinoids on Growth of Human LNCaP Prostate Cancer Cells and Tumor Xenografts. Bioorg Med Chem 16, 3352–3360 (2008). 18166465 10.1016/j.bmc.2007.12.007 PMC2374748

[b33] Pinto M. . Enterocytic differentiation of cultured human colon cancer cells by replacement of glucose by galactose in the medium. Biol. Cell 44, 193–196 (1982).

[b34] Cunnane G. . Quantitative analysis of synovial membrane inflammation: a comparison between automated and conventional microscopic measurements. Ann. Rheum. Dis. 58, 493–499 (1999). 10419868 10.1136/ard.58.8.493 PMC1752933

