# Supplementary material for: Potent Inducers of Endogenous Antimicrobial Peptides for Host Directed Therapy of Infections
Source: Sci Rep. 2016 Nov 9;6:36692. doi: 10.1038/srep36692 (PMC5101518; doi:10.1038/srep36692)

SUPPLEMENTARY INFORMATION
to

Potent Inducers of Endogenous Antimicrobial Peptides for Host Directed Therapy of Infections.

H. Ottosson, F. Nylén, P. Sarker, E. Miraglia, P. Bergman, G. H. Gudmundsson,
R. Raqib, B. Agerberth & R. Strömberg*.

CONTENTS:

S 1: Comparison of induction in the reporter cell line with and without pre-differentiation of cells with galactose p. 2

S 2: Inducing ability vs HDAC inhibitory data for APD compound **5** (Entinostat) vs the potent HDAC inhibitors Vorinostat and Trichostatin A p. 3

S 3: Induction with compound **19** that has a more flexible non-benzoyl amide linked to the phenylenediamine p. 4

S 4: Bacterial count from three recovering rabbits upon administration of a 2mg single dose of (**5**, Entinostat). p. 4

S 5: Immunohistochemical staining of CAP-18 in sections of colon from healthy rabbits and a rabbit model of Shigellosis, at two different magnifications. p. 5

S 6: Schemes for synthesis of compounds. p. 6

S 1: Comparative fold induction assays in the MN8CampLuc reporter cell line with and without pre-differentiation of cells by treatment with galactose.

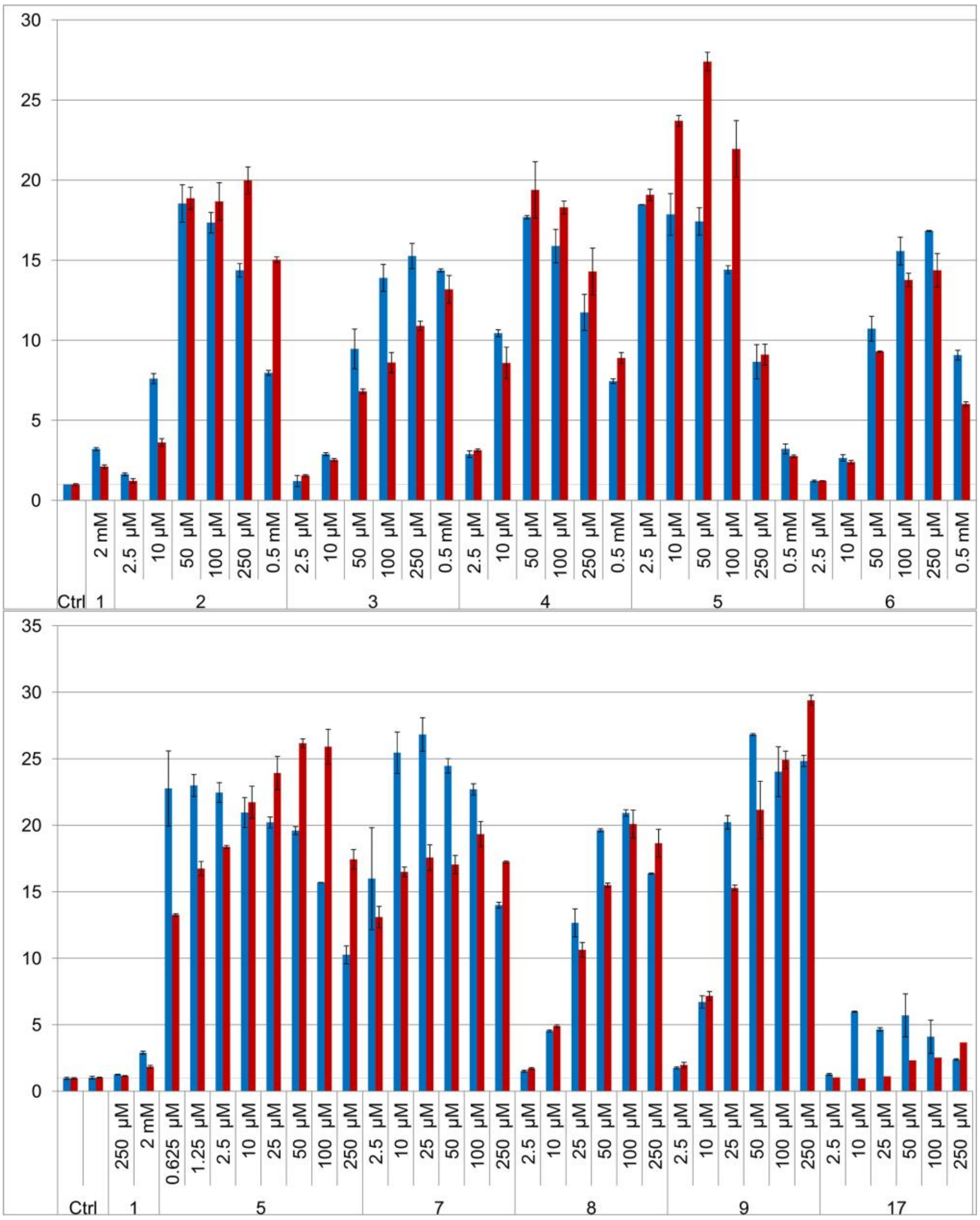

Figure S1. Graphs of fold induction (as luciferase activity) in the MN8CampLuc reporter cell line as elicited by APD's **2**-**9** with or without pre-differentiation of the cells with galactose. (red columns: data from pre-differentiated cells; Blue columns: data from non pre-differentiated cells). Error bars represent standard deviation. The fold induction values are relative to the background from untreated cells and all experiments were performed in duplicate (triplicate for some). Bars for standard deviation are shown and all differences with compounds **2**-**9** relative to untreated cells were statistically significant (Student's t-test p-values were all <0.01 except for 2.5 µM of **2** in prediff. cells (upper graph, red bar, p-value, 0.025)) except for 2.5 µM of **3** (upper graph, red bar, p-value, 0.60, blue bar, p-value 0.12). Apart from at the two highest concentrations of 17 the difference to untreated cells were not significant for prediff. cells (p-values: 0.16, 0.18, 0.24 and 0,08 for 2.5, 10, 25 and 50 µM, respectively). With non-prediff. cells (blue bars) the differences were however significant (p-values < 0.01) which may suggest that a major reason for the induction with **17** is its ability to differentiate the cells and representative for the extent of induction obtained by only HDAC inhibition.

S 2: Induction *vs* HDAC inhibition.

The potent HDAC inhibitors Vorinostat (**17**) and Trichostatin A (**18**) give considerably lower fold induction than compound **5** (Figure S2) although all known IC_{50} values for HDAC inhibition shows that compounds **17** and **18** are considerably more powerful HDAC inhibitors (Table S1).

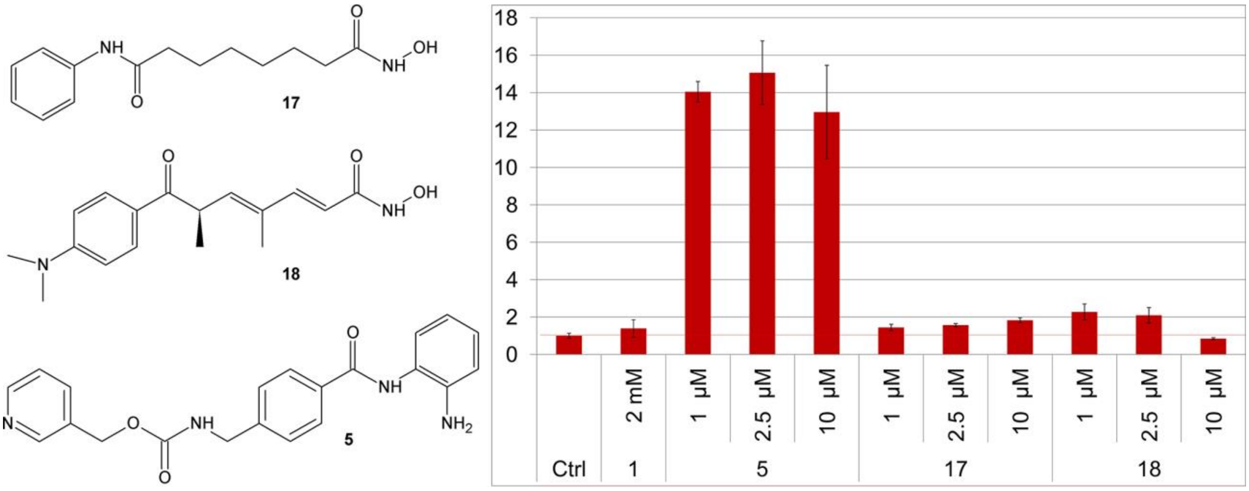

Figure S2. To the left: structures of APD-compound **5** (Entinostat) as well as of Vorinostat (**17**) and trichostatin A (**18**). Graph to the right: Fold induction (as luciferase activity) assays in the MN8CampLuc reporter cell line with compound **5** compared to treatment with the known HDAC inhibitors Vorinostat (**17**) and Trichostatin A (**18**) (data was obtained from cells that were pre-differentiated by treatment with galactose). Error bars represent standard deviation. The fold induction values are relative to the background from untreated cells and all experiments were performed in duplicate (triplicate for some). The differences compared to the control are significant for all concentration of **5** (p values, 0.011 or lower) but not for highest concentration of **18** (p-value, 0.014) and barely for the lowest concentration of **17** (p-value, 0.055). For higher conc of **17** there was a small significant increase compared to the control (p-values: 0.02 and 0.013 for 2.5 and 10 µM respectively). For lower concentration of **18** there was also a small significant increase compared to control (p-values: 0.029 and 0.036 for 1 and 2.5 µM respectively). The differences between compounds **5** and **17** or **18** were highly significant at all concentrations (p-values < 0.012 or lower).

Table S1. Table with IC50 values [µM][a] for HDAC inhibition of different histone deacetylases by compounds **5**, **17** and **18**.

Compound	**5**		**17**		**18**	
	#	Avg ±stdev	#	Avg ±stdev	#	Avg ±stdev
HDAC1	*24*	*0.36 ±0.29*	*85*	*0.11 ±0.11*	*43*	*0.014 ±0.028*
HDAC2	*9*	*0.36 ±0.27*	*49*	*0.14 ±0.12*	*27*	*0.013 ±0.014*
HDAC3	*9*	*0.43 ±0.17*	*40*	*0.08 ±0.06*	*9*	*0.006 ±0.004*
HDAC4	*5*	*41 ±53*	*7*	*1.2 ±2.0*	*16*	*0.047 ±0.049*
HDAC5	*1*	*1.4*	*11*	*6.6 ±6.8*	*5*	*0.020 ±0.018*
HDAC6	*1*	*13*	*59*	*0.062 ±0.058*	*29*	*0.036 ±0.055*
HDAC7	*1*	*>100*	*7*	*15 ±17*	*7*	*0.034 ±0.043*
HDAC8	*3*	*21 ±18*	*40*	*1.2 ±0.7*	*16*	*0.453 ±0.513*
HDAC9	*1*	*>100*	*5*	*3.2 ±6.2*	*4*	*0.029 ±0.009*
HDAC10	*1*	*11*	*9*	*0.19 ±0.15*	*9*	*0.011 ±0.006*
HDAC11	*0*	*-*	*5*	*0.26 ±0.14*	*5*	*0.018 ±0.006*
HDAC[b]	*14*	*3.5 ±2.0*	*93*	*0.23 ±0.28*	*32*	*0.058 ±0.105*

[a] The average IC_{50} values from all reported assays in PubChem (2014-07-08) was used. Assays with any data from specific compounds deviating more than 2 standard deviations from the average, were excluded. b HDAC type not specified in the assay. # Number of assays included in the dataset.

S 3: Induction with compound **19** that has a more flexible non-benzoyl amide linked to the phenylenediamine.

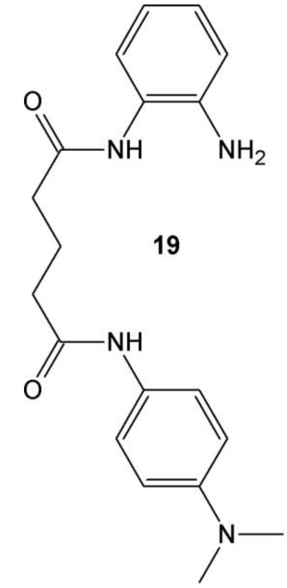

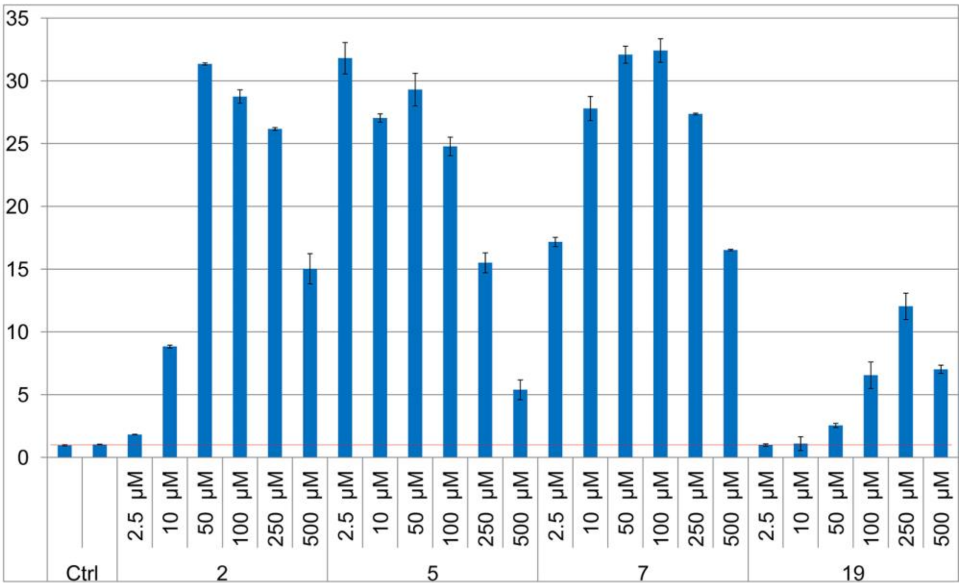

Figure S3. Comparison of induction with compounds **2**, **5** and **7** with that of compound **19** that has a more flexible non-benzoyl amide linked to the phenylenediamine => reduced induction. Error bars represent standard deviation. The fold induction values are relative to the background from untreated cells and all experiments were performed in duplicate (triplicate for some). The differences compared to the control are significant for all concentrations of **2**, **5** and **7** and for 50-100 μM of **19** (p values, < 0.01) but not for 2.5 and 10 μM of **19** (p-values: 0.43 and 0.098 respectively).

S 4: Bacterial count from three recovering rabbits upon administration of a 2mg single dose of pyridin-3-ylmethyl (4-((2-aminophenyl)carbamoyl)benzyl)carbamate (**5**, Entinostat).

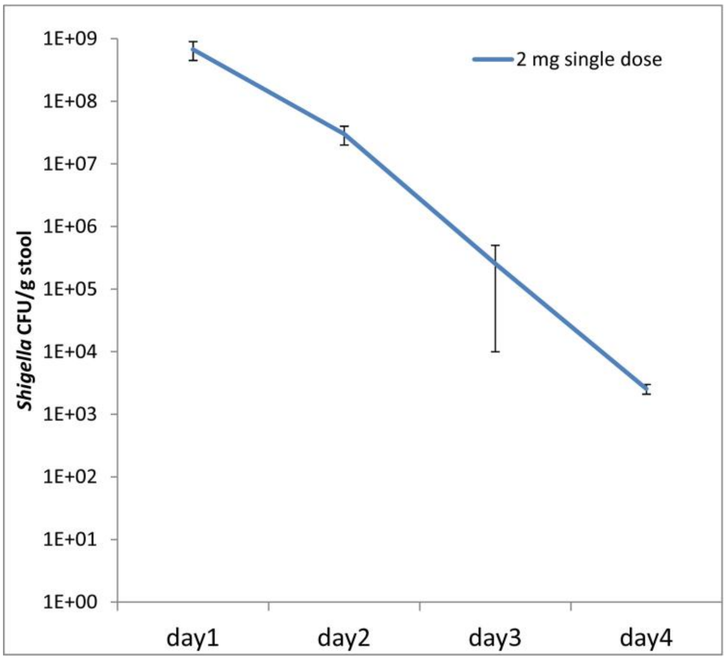

Figure S4. Graph of bacterial count from stool of *Shigella* infected rabbits after treatment with a single 2mg dose of **5** (average from three rabbits since one of the five was weak and sacrificed and one died in the first night, one rabbit was also not included for day 1 and 2 since colonies were uncountable due to contamination on day three and four). Error bars represent spread of count values for the different rabbits. The reduction in counts from day one to four is not statistically significant (paired t-test p-value, 0.2), partially because not enough rabbits survived or could give countable colonies, but the trend is similar to what was found with the lower doses.

S 5: Immunohistochemical staining of CAP-18 in sections of colon from healthy rabbits and a rabbit model of Shigellosis, at two different magnifications.

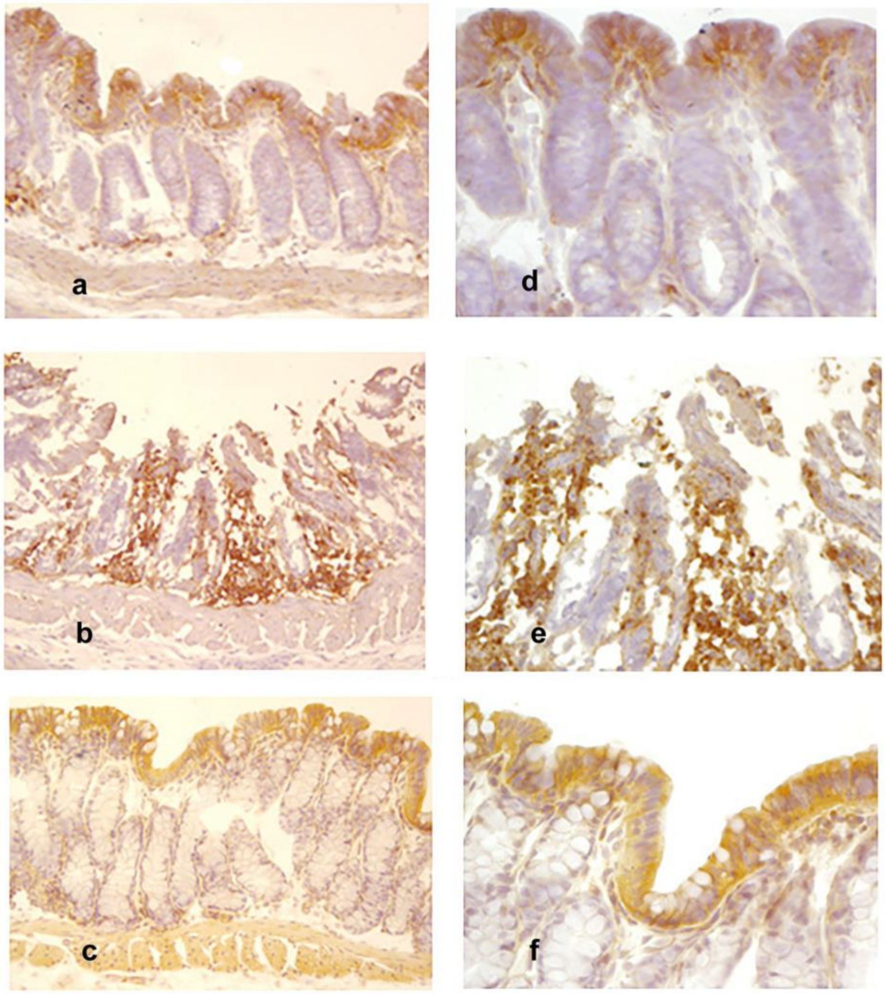

Figure S5: Immunohistochemical staining of CAP-18 in sections of rabbit colon visualized at 200 x (**a**-**c**) and 400 x (**d**-**f**) magnification. **a** and **d**: Immunoreactive signals for CAP-18 (brown) in healthy rabbits were almost exclusively located in the surface epithelium. **b** and **e**: In Shigella-infected rabbits, epithelial surface was almost devoid of CAP-18 staining and abundant CAP-18-expressing inflammatory cells were seen in the lamina propria. **c** and **f**: Reappearance of CAP-18 staining in the surface epithelium and disappearance of CAP-18 expressing cells from the lamina propria in infected rabbits treated with 0.5 mg doses (twice daily for two days) of pyridin-3-ylmethyl (4-((2- aminophenyl)carbamoyl)benzyl)carbamate (**5**, Entinostat).

S 6. Schemes for synthesis of compounds.

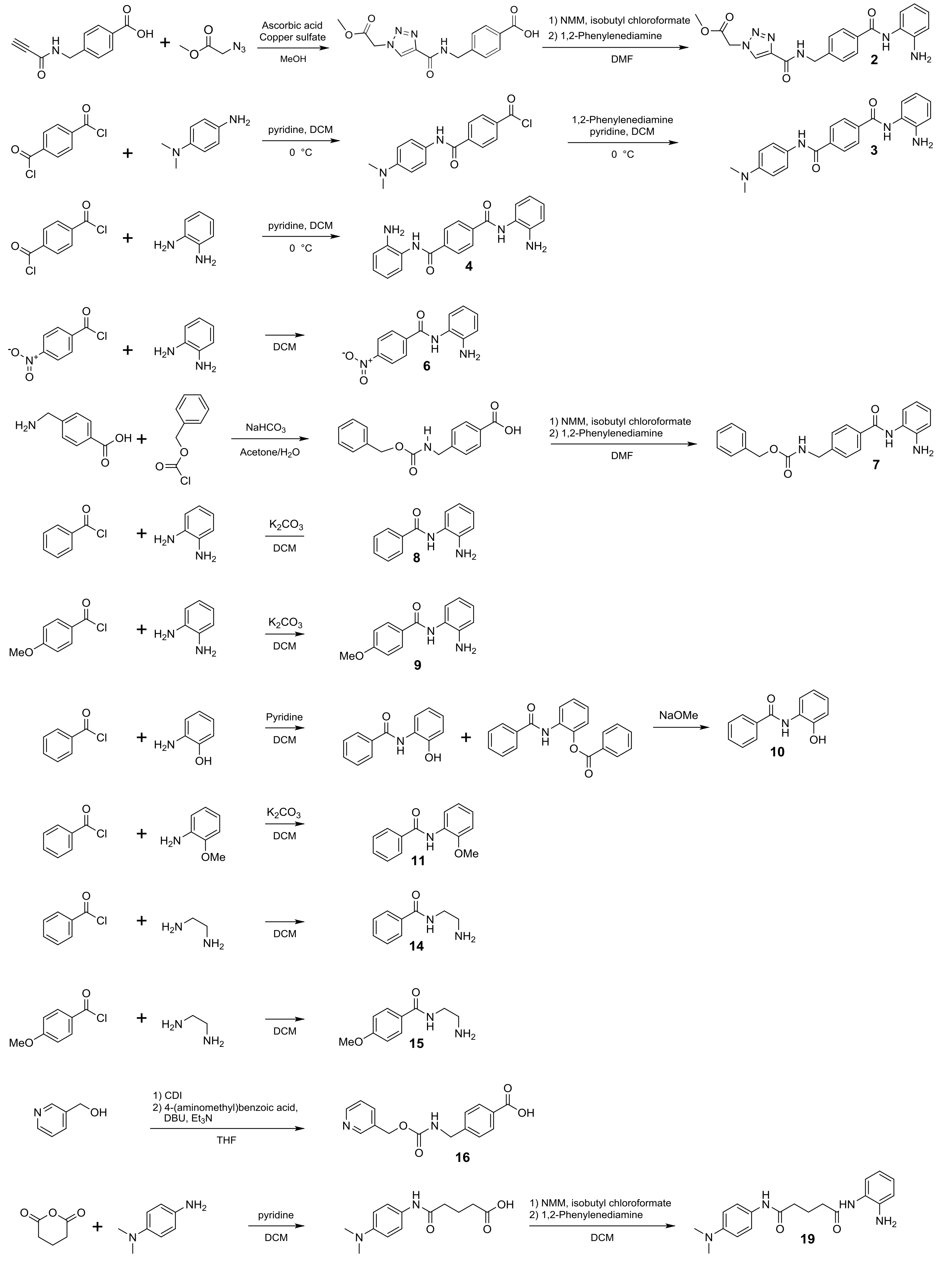

Supplement: Supplementary Information [file srep36692-s1.pdf]
